# Comparative assessment of methods for the fusion transcripts detection from RNA-Seq data

**DOI:** 10.1038/srep21597

**Published:** 2016-02-10

**Authors:** Shailesh Kumar, Angie Duy Vo, Fujun Qin, Hui Li

**Affiliations:** 1 Department of Pathology, School of Medicine, University of Virginia, Charlottesville, VA 22908; 2 Department of Biochemistry and Molecular Genetics, School of Medicine, University of Virginia, Charlottesville, VA 22908.

## Abstract

RNA-Seq made possible the global identification of fusion transcripts, i.e. “chimeric RNAs”. Even though various software packages have been developed to serve this purpose, they behave differently in different datasets provided by different developers. It is important for both users, and developers to have an unbiased assessment of the performance of existing fusion detection tools. Toward this goal, we compared the performance of 12 well-known fusion detection software packages. We evaluated the sensitivity, false discovery rate, computing time, and memory usage of these tools in four different datasets (positive, negative, mixed, and test). We conclude that some tools are better than others in terms of sensitivity, positive prediction value, time consumption and memory usage. We also observed small overlaps of the fusions detected by different tools in the real dataset (test dataset). This could be due to false discoveries by various tools, but could also be due to the reason that none of the tools are inclusive. We have found that the performance of the tools depends on the quality, read length, and number of reads of the RNA-Seq data. We recommend that users choose the proper tools for their purpose based on the properties of their RNA-Seq data.

Nowadays, RNA-Sequencing technology^1^ is playing an important role in characterizing the whole transcriptome in any given sample. Quantification of gene expression, identification of novel transcripts, and detection of fusion transcripts are the major applications of RNA-Seq. In humans, fusion transcripts, also known as “chimeric transcripts”, can been generated by several mechanisms^2^,^3^. Traditionally, fusion RNA detection has facilitated the detection, and diagnosis of various tumors^4^,^5^,^6^,^7^,^8^. More recently, fusion transcripts have also been found in non-neoplastic tissues^9^,^10^. Identification of fusion genes from RNA-Seq data can be accomplished with the help of different software packages, which are freely available to the scientific community. These software packages have been trained, and tested on different types of datasets, and follow different algorithms. Even though that in the past six years, around 20 tools have been developed for the detection of fusion transcripts from RNA-Seq data, there are still various challenges associated with these tools. An ample amount of time and computational power are required for these software packages to function. Behavior exhibited by these tools changes with datasets. In addition to missing true fusion events, they can also produce false positives^11^. So, there is a need for practical knowledge of these tools in terms of time consumption, computational memory usage, sensitivity, and specificity. For this purpose, the comparison of all fusion detection tools should be performed on unbiased datasets.

In the past, some attempts have been made to compare these software packages. In 2013, Carrara *et al.*^11^ compared the performance of six fusion detection software tools (i.e. FusionHunter^12^, FusionMap^13^, FusionFinder^14^, MapSplice^15^, defuse^16^, and TopHat-Fusion^17^) with positive and negative datasets. However, several newly developed tools like BreakFusion^18^, SOAPfuse^19^, JAFFA^20^, nFuse^21^, EricScript^22^, and FusionCatcher^23^ were not included in this study. Even though some comparisons were included in the papers from the developers of these new software tools, there has been no unbiased study published to compare the fusion detection rate (positive and negative), time consumed, and computational power utilized by all of the tools.

In this study, we have gone through all of the fusion detection software packages available to date (~20), and accessed the performance of the 12 best tools (Table 1). These tools have been compared on four types of datasets, 1) positive dataset: a dataset of simulated reads, containing 50 positive fusion sequences; 2) negative dataset: simulated dataset, consist of reads from 6 libraries, provided by Carrara *et al.*^11^; 3) mixed dataset: we prepared this dataset by combining the reads of the positive dataset, and a library (i.e. Lib_75_R1) of the negative dataset; 4) Test dataset: a six RNA-Seq run dataset, that we had previously analyzed, with a total of 44 fusions successfully confirmed by Sanger’s sequencing^10^. Performance of these tools has been compared in terms of detected fusions, sensitivity, positive prediction values, computational memory (i.e. RAM), and time consumption for all the datasets. Finally, we performed a TOPSIS^24^ (Technique for Order of Preference by Similarity to Ideal Solution) analysis on the mixed dataset results, and ranked the fusion detection tools. Detailed comparisons of the performance, and limitations of each tool for a particular dataset are discussed.

## Methods

### Fusion detection software packages

Bellerophontes, BreakFusion, Chimerascan, nFuse, EricScript, FusionCatcher, FusionHunter, FusionMap, JAFFA, MapSplice, SOAPfuse, and TopHat-Fusion packages were downloaded and installed on our server (http://uvacse.virginia.edu/resources/rivanna/rivanna). All of the software packages were run using a default configuration of each tool with SLURM (Simple Linux Utility for Resource Management) scripts. Brief descriptions of each fusion detection tool are given in this section. A summary of these tools is included in Table 1.

#### Bellerophontes

developed by Abate *et al.* 2012^25^, is a software package that detects fusion transcripts from short paired-end reads by implementing JAVA and Perl. It integrates “splicing-driven alignment” and “abundance estimation analysis”, to generate a more accurate set of reads supporting the junction discovery. The transcripts, which are not annotated, are also taken into account. Bellerophontes selects the putative junctions on the basis of a match with an accurate gene fusion model^25^. Here, we used Bellerophontes version 0.4.0.

#### BreakFusion

Is a pipeline using one, or a set of whole transcriptome BAM files, with mapped-paired end RNA-Seq reads to detect gene fusion candidates in five steps. First, splicing breakpoints are identified by using a read-pair algorithm, or a splice mapping algorithm. Then, shorts reads anchored around each breakpoint are locally constructed using TIGRA^26^. This creates a set of splice junction contigs, which are supported by mapped, and one-end anchored reads. Step three involves the use of BLAT^27^ to align junction sequences to the genome. Then the BLAT alignments are summarized into a chimeric score, that numerically represents the probability of an assembled junction sequence having bona fide points relative to the genome. Step five involves breakpoint annotation using UCSC databases^28^.

#### Chimerascan

Developed using Python programming language, uses Bowtie to align paired-end reads with a merged genome-transcriptome reference^29^. A combined index is formed from FASTA sequences of genome and transcript features (UCSC GenePred format) files. Subsequent steps after the alignment are; 1) trimming of the alignment, 2) identification of discordant reads, 3) nomination of chimeras, 4) junction alignment, and 5) final chimera identification^29^. We used Chimerascan version 0.4.5 for this study.

#### EricScript

(chimEric tranScript detection algorithm)^22^ is a Perl based tool, using R^30^, ada^31^, BWA^32^, SAMtools^33^, Bedtools^34^, seqtk, and BLAT for the identification of chimeric transcripts. It comprises the following steps; 1) Mapping of RNA-Seq reads to the reference transcriptome, 2) Identification of disputatious (i.e. discordant) alignments, and construction of exon junction reference, 3) Recalibration of exon junction references, and 4) Scoring and filtering the candidate gene fusions. EricScript version 0.5.1 was used for this study.

#### FusionCatcher^23^

is a Python based tool, using Bowtie^35^, Bowtie2^36^, BWA, BLAT, Liftover, STAR^37^, Velvet^38^, Fatotwobit, SAMtools, Seqtk, Numpy, Biopython, Picard, and Parallel for fusion identification. Here, we used FusionCatcher_v0.99.4c.

#### FusionHunter^12^

is a Perl based tool, using Bowtie to align the paired-end reads against a reference genome. Mapped reads are then used to detect fusions, which are collected to make a pseudo reference. Unmapped reads are broken and aligned on this pseudo reference. If one broken portion is correctly aligned, the nearest recognized splicing junction is searched, and the alternate part of the mother read is aligned to this region. FusionHunter also uses several strategies to discard false fusions. FusionHunter identified only fusion transcripts with junction sites at the exon edge (splicing junction), but it could not detect a fusion transcript with junction sites in the middle of an exon. FusionHunter-v1.4-Linux_x86_64 was used for this study.

#### JAFFA^20^

is the latest pipeline that we used for this benchmark study. It uses several external softwares, mainly Bpipe, Velvet, Oases^39^, SAMtools, Bowtie2, BLAT, Dedupe, Reformat, and R packages, for the detection of fusions. This pipeline runs in three modes: 1) ‘assembly’ mode, which assembles the short reads into transcripts before fusion detection; 2) ‘direct’ mode, which uses reads that do not map to known transcripts; 3) ‘hybrid’ mode, which both assembles transcripts, and supplements all of the assembled transcript contigs with unmapped reads^20^. The appropriate mode to use depends upon the length of RNA-Seq reads. Assembly mode must be used for reads having lengths less than 60bp. Reads having lengths 60bp to 99bp should be analyzed by hybrid mode. Direct mode should be used for the reads having lengths of 100bp or more. JAFFA requires reference transcripts from GENCODE^40^. We used JAFFA-version-1.06.

#### MapSplice^15^

is a software package developed in the Python programming language. The MapSplice algorithm works in several steps. First, it splits each read into a set of consecutive elements, and then exon alignment is performed. By using the knowledge of other aligned elements, it aligns the elements, which are not aligned in the previous step. Second, it uses two statistical measures to check the quality of the splice junctions identified in the first step. These two measures are: 1) “anchor significance”, produced by an alignment of maximum significance, resulting from long anchors on the both sides of splice junctions, and 2) “entropy”, which is calculated by the multiplicity of splice junction locations^15^. For fusion detection, MapSplice uses a prebuilt Bowtie index of the human genome, prebuilt gene annotation files in GTF format, and human chromosome files in FASTA format. For this study, we used MapSplice-v2.1.9.

#### FusionMap^13^

is a windows-based tool, using Mono^41^ to run on the Linux platform. It splits the reads into small fragments, and aligns those to annotated genes. This alignment of reads is based on an algorithm known as GSPN^13^, which provides an acceptance of at most two bases. To refine the position of junction boundaries, all chimeras having fusion boundary distances less than 5 bp are combined. Established splicing patterns are also used to refine the site of the fusion boundary. Several filters are used to remove false positive fusions. Here, FusionMap_2015-03-31 version was used.

#### nFuse^21^

is a Perl based standalone package, which also uses some Python and R scripts. External software like BLAT, Bowtie, Bowtie2 and Gmap are also required to run nFuse. nFuse is the advance version of deFuse, using both genome and transcriptome sequencing reads. It requires pre-built Bowtie references, transcriptome files (both GTF and FASTA format), EST files (FASTA format), genome files (FASTA format), and Gmap references. Here, we used deFuse script of nFuse version 0.2.1.

#### TopHat-Fusion^17^

uses two scripts (“Tophat” and “Tophat-fusion-post”) for the complete analysis of fusion candidates. It detects fusions by performing several steps: 1) creating partial exons from the alignment, generated by mapping of reads to exons, 2) generation of pseudo-genes, while unmapped reads are split into shorter elements, and mapped on the genome, 3) detection of chimeras, if reads fragments map in a steady way with fusions, and 4) filtering to eliminate chimeras associated with multi-copy genes, or repetitive sequences^17^. Tophat-2.1.0.Linux_x86_64 version was used for this study.

#### SOAPfuse^19^

is a standalone package, developed in Perl. It uses a pre-built database, including whole genome and transcriptome indexes. It combines the alignment of RNA-Seq paired-end reads against the annotated genes, and human genome reference as well. SOAPfuse pursues two types of reads to support a fusion event: 1) span-reads, discordant mapping paired-end reads connecting the candidate fusion gene pairs, and 2) junction-reads, that conform to the exact junction sites. We used SOAPfuse-v1.26 version of this software.

### Datasets

#### Positive dataset

The positive dataset contains a total of 57,209 synthetic pairs of reads (i.e. paired-end), having 75nt lengths with 158bp fragment lengths. This dataset was generated by the FusionMap^13^ developers. It contains a total of 50 true fusions, supported by read pairs ranging from 9 to 8,852.

#### Negative dataset

We used the same negative dataset used by Carrara *et al.*^11^. This dataset consists of six sets (three sets in duplicates) of paired-end reads with read-lengths of 50nt (Lib50_1 and Lib50_2), 75nt (Lib75_1 and Lib75_2), and 100nt (Lib100_1 and Lib100_2) respectively. Initially, two different quality score libraries (i.e. Lib100_1 and Lib100_2) were developed by BEERS^42^. Afterwards, 50nt sets (Lib50_1 and Lib50_2) and 75nt sets (Lib75_1 and Lib75_2) were prepared by trimming 50nt and 25nt from the beginning of Lib100_1 and Lib100_2 respectively. Construction of this dataset is described in the article published by Carrara *et al.*^11^.

#### Mixed dataset

A mixed dataset was prepared by combining 70,000,000 pair reads of Lib75_1 from the negative dataset and 57,209 pair reads from the positive dataset. The length of all reads in the mixed dataset was 75nt.

#### Test dataset

We used six sets of Illumina HiSeq 2000 paired-end RNA-Seq reads from our previous study^10^. The NCBI accession numbers of all six runs are SRR1657556, SRR1657557, SRR1657558, SRR1657559, SRR1657560, and SRR1657561 respectively. Prior to analysis, we filtered the raw reads with the NGS QC toolkit^43^. Finally, totals of 62,117,396 (i.e. SRR1657556), 58,060,054 (i.e. SRR1657557), 7,444,600 (i.e. SRR1657558), 7,463,410 (i.e. SRR1657559), 7,294,844 (i.e. SRR1657560), and 7,291,426 (i.e. SRR1657561) high quality, vector/adaptor filtered, paired-end reads were used for this study. We called “larger data” to SRR1657556 (100nt read length) and SRR1657557 (100nt read length) runs, and “smaller data” to SRR1657558 (50nt read length), SRR1657559 (50nt read length), SRR1657560 (50nt read length) and SRR1657561 (50nt read length) runs.

### Data analysis

We ran all 12 tools at default parameters, and analyzed the performance of each tool using each dataset (i.e. positive, negative, mixed, and test). For each run of every dataset, we calculated the computational memory used (GB), and time consumed (minutes). We manually checked the identified fusion genes in all of the results produced by each tool with each dataset. We used the human hg19 database as a reference sequence.

We used the following parameters to assess the sensitivity and specificity of the tools.

1. Sensitivity (%) = (TP/TF) * 100
2. Positive predictive value (PPV) (%) = (TP/TP+FP) * 100

TP: - True positive. Correctly identified fusions.

TF: - Total fusions.

FP: - False positive.

TOPSIS (Technique for Order of Preference by Similarity to Ideal Solution)^24^ analysis was performed to make decisions on the basis of multiple criteria results for each tool. We used the mixed dataset results for TOPSIS analysis, in order to rank each software package. The methodology with an example is described here (http://hodgett.co.uk/topsis-in-excel/). For each tool, TOPSIS scores were calculated by taking two types of weights for all of the four criteria i.e. sensitivity, time consumption (minutes), computational memory (RAM), and PPV. We compared the performance of the tools under two scenarios. In the first scenario, we equally weighted all of the four criteria (i.e. weight for each criteria is 0.25). In the second, we decided to give more weight to sensitivity and PPV (i.e. 0.35 for both), and less weight to time and computational memory consumption (i.e. 0.15 for both). TOPSIS scores were calculated separately for both cases.

## Results

For this benchmark study, we analyzed a total of 20 software packages currently available. For various reasons we failed to obtain, or run eight of them, which resulted in the analysis of 12 software packages. We attempted using the FusionFinder^14^ software package, which was last updated for Perl API, and Ensembl version 68. However, the Ensembl server located in the USA (useastdb.ensembl.org) does not have version 68. We then tried using the UK server (ensembldb.ensembl.org), and found that running FusionFinder from the UK server took an incomprehensible amount of time when compared to the other software packages. IDP-fusion^44^ is a hybrid fusion detection software tool, designed to run for long reads (product of third generation sequencing technologies) mixed with short reads, which does not fit the purpose of this study. McPherson *et al.* developed three fusion detection software packages i.e. Comrad^45^, deFuse^16^ and nFuse^21^. Comrad is the oldest software in this group, and is no longer maintained. nFuse software contains both deFuse and nFuse scripts for fusion detection. The nFuse script of the package is used only for a combination of long reads and short reads. Therefore, we used the defuse script of the nFuse software package. FusionAnalyser^46^ and FusionMap^13^ are windows-based software packages, which run on the Linux system, with the help of Mono. We were unable to run FusionAnalyser on our server because of compatibility issues, but runs with FusionMap were successful. We could not locate a copy of the SnowShoes-FTD pipeline on the ftp site reported previously^47^.

### Positive dataset

**Figure 1:**
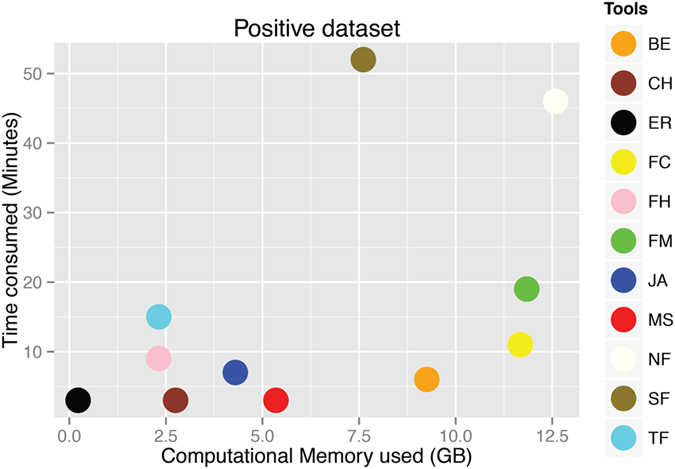
Comparison of time and computational memory used by software packages on the positive dataset. **BE**: Bellerophontes, **CH**: Chimerascan, **ER**: EricScript, **NF**: nFuse, **FC**: FusionCatcher, **FH**: FusionHunter, **FM**: FusionMap, **JA**: JAFFA, **MS**: MapSplice, **SF**: SOAPfuse, **TF**: TopHat-Fusion.

All 12 tools were used to analyze a positive dataset of 57,209 paired-end reads. This dataset contains 50 true fusions. Results in terms of true fusions detected, time comsumed, and computational memory used are reported in Table 2. Only Bellerophontes detected false fusions with this dataset. Out of the 42 fusions predicted by Bellerophontes, nine are false positives, and 33 are true fusions. For this dataset, JAFFA is the most sensitive tool. Based on sensitivity, the tools can be ordered as follows: JAFFA (88%) > MapSplice (86%) > SOAPfuse (82%) > EricScript (78%) > FusionCatcher (66%) = Bellerophontes (66%) > FusionMap (56%) > TopHat-Fusion (54%) > FusionHunter (36%) > nFuse (30%) > Chimerascan (8%) > BreakFusion (4%). Comparisons between time consumed (minutes) and computational memory (i.e. RAM) used by the tools indicated that, EricScript is the most efficient tool (Fig. 1), consuming ~0.228 GB of computational memory (i.e. RAM) for only three minutes (Table 2). Other efficient performers include Chimerascan, FusionHunter, and TopHat-Fusion (Fig. 1), but they suffer from poor sensitivity values i.e. 8%, 36%, and 54% respectively. nFuse is the least efficient tool in terms of computational memory usage and time consumption, consuming ~12.6 GB of RAM for 46 minutes., as shown in (Table 2). JAFFA, the best performer in terms of sensitivity (i.e. 88%) has a descent balance between time consumption and computational memory usage, using 5.35 GB of RAM for three minutes. The time consumption and memory usage of BreakFusion has not been calculated because it starts with a prebuilt BAM file. Thus, it is not fair to compare it with the other 11 tools that start with FASTQ files.

### Negative dataset

All of the 12 tools were run on the 6-library dataset (i.e. Lib_50_R1, Lib_50_R2, Lib_75_R1, Lib_75_R2, Lib_100_R1, and Lib_100_R2), which represet the negative dataset. BreakFusion, SOAPfuse, and EricScript runs were not completed due to software errors occurring in the handling of intermediate files. Chimerascan, only finished Lib_50_R2, and yielded false fusion discoveries. FusionCatcher had the lowest false discovery rate. For all six libraries, this tool found zero false fusions. For Lib_50_R1, FusionMap and nFuse identified a total of 93, and five false fusions respectively (Supplementary Table S1). The rest of the tools did not detect any fusions. Most of the software packages detected fusions for Lib_50_R2. A total of 15,465, 1,600, 842, 27, 51, 152, 70, and 29 fusions were produced by Bellerophontes, Chimerascan, FusionHunter, FusionMap, JAFFA, MapSplice, nFuse, and TopHat-Fusion respectively (Supplementary Table S1).

**Figure 2:**
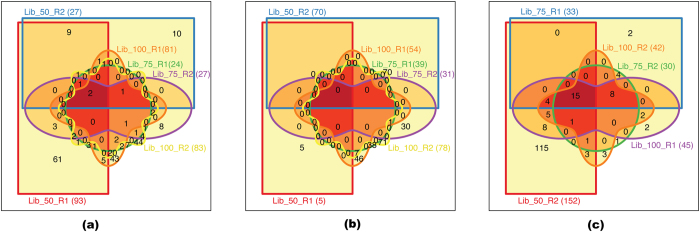
Common false fusions present in the negative dataset. (**a**) FusionMap, (**b**) nFuse and (**c**) MapSplice.

The number of false fusions identified tends to increase with the read length of the dataset, when comparing 75bp reads with 100bp reads (Fig. 2). FusionMap (Fig. 2(a)) identified a total of 24 and 27 fusions for Lib_75_R1 and Lib_75_R2 respectively. For Lib_100_R1 and Lib_100_R2, the number of fusions increased to 81 and 83. The same trend was observed with nFuse (Fig. 2(b)), and MapSplice (Fig. 2(c)). However, the trend became complicated when Lib_50 libraries were considered. In this situation, both the read length, and the quality scores of the reads may contribute to the false discovery rates. In the case of Lib_50_R2, most software tools generated a drastic increase in the number of false fusions (Supplementary Table S1). Presumably, this is due to a lower quality score of this library, as compare to Lib_50_R1^11^.

Figure 2 shows the venn diagrams of the comparison of false fusions, detected by FusionMap (Fig. 2(a)), nFuse (Fig. 2(b)), and MapSplice (Fig. 2(c)) on all six libraries of the negative dataset. It shows the unique and shared fusion transcripts among different libraries of a particular tool. FusionMap found a total of 61, 10, 7, 8, 43, and 44 unique fusions from Lib_50_R1, Lib_50_R2, Lib_75_R1, Lib_75_R2, Lib_100_R1, and Lib_100_R2 respectively. nFuse found 5, 70, 38, 30, 46, and 71 unique fusions. MapSplice detected a total of 115, 2, 1, 2, and 3 unique fusions from Lib_50_R2, Lib_75_R1, Lib_75_R2, Lib_100_R1, and Lib_100_R2 libraries respectively. A very small number of false fusions were detected in all libraries using any of the three software tools (2 for FusionMap, 0 for nFuse, and 15 for MapSplice).

For all six libraries of the negative dataset, a comparison of fusion detection tools in terms of time consumption and memory usage is shown in the Supplementary Fig. S1. FusionMap had the best balance among the tools in all cases. It consumed 12.41GB for 105 minutes, 11.56 GB for 81 minutes, 12.08 GB for 113 minutes, 12.58 GB for 111 minutes, 11.56 GB for 130 minutes, and 11.57 GB for 136 minutes to analyze Lib_50_R1, Lib_50_R2, Lib_75_R1, Lib_75_R2, Lib_100_R1, and Lib_100_R2 respectively (Supplementary Sheet S1). Bellerophontes and FusionHunter performed almost equally using the negative dataset, and lie at the second tier (Supplementary Fig. S1). For all six libraries of the negative dataset, performance of JAFFA was poor compared to other tools. It consumed 85.90 GB for 3,045 minutes, 49.49 GB for 1,446 minutes, 89.58 GB for 2,305 minutes, 90.62 GB for 2,497 minutes, 91.64 GB for 3,608 minutes, and 91.61 GB for 3,460 minutes to analyze the data of Lib_50_R1, Lib_50_R2, Lib_75_R1, Lib_75_R2, Lib_100_R1, and Lib_100_R2 respectively (Supplementary Sheet S1).

### Mixed dataset

A typical paired-end RNA sequencing dataset nowadays contains 50–100 million reads. The positive dataset we tested above only has 57,209 paired-end reads. To compare the software tools in a relatively realistic setting, we decided to mix the positive dataset with the Lib75_1 (containing 70 million paired-end reads) from the negative dataset. The length of all reads in the mixed dataset was 75nt. For this dataset, BreakFusion, Chimerascan, and SOAPfuse did not complete because of the unavailability of a significant amount of supporting reads at the intermediate steps, which resulted in error messages. FusionHunter finished the run, but was unable to detect any fusions. Based on sensitivity, the tools can be ordered as follows: MapSplice (84%) > EricScript (78%) > nFuse (76%) > FusionMap (72%) > Bellerophontes (68%) > FusionCatcher (62%) > TopHat-Fusion (56%) > JAFFA (44%) (Table 3). EricScript, FusionCatcher, and TopHat-Fusion did not detect any false positive fusions, i.e. all the fusions predicted by these tools were true fusions. On the basis of Positive Prediction Values (PPV), the tools can be ordered as follows: EricScript (100%) = FusionCatcher (100%) = TopHat-Fusion (100%) > JAFFA (95.6%) > nFuse (95%) > Bellerophontes (79%) > FusionMap (60%) > MapSplice (54%) (Table 3). These results suggest that although MapSplice detected the maximum number of true fusions, it also identified a large number of false fusions. EricScript has the best balance between true and false fusion detection. It detected a total of 39 out of 50 true fusions, and no false fusions. In terms of memory uses and time consumed in analyzing this mixed dataset, the performances of EricScript, FusionCatcher, FusionMap, Bellerophontes, and FusionHunter are better than other tools. EricScript used 4.67 GB for 677 minutes (Supplementary Fig. S2). FusionMap used 12.50 GB for 120 minutes. nFuse and TopHat-Fusion ranked in the second tier. MapSplice consumed 5.48GB of RAM for 3,825 minutes (Supplementary Fig. S2). Similar to the negative dataset, the performance of JAFFA is poor using the mixed dataset, using 89.4 GB of RAM for 3,845 minutes.

### Test dataset

A set of six RNA-Seq runs (i.e. SRR1657556, SRR1657557, SRR1657558, SRR1657559, SRR1657560, and SRR165761) representing the test dataset was also used to assess the performance of fusion detection tools. RNA-Seq runs SRR1657558, SRR1657559, SRR1657560, and SRR165761 have 50nt read lengths, and have a total of 7,444,600, 7,463,410, 7,294,844, and 7,291,426 paired-end reads respectively, representing “smaller data”. FusionCatcher, FusionMap, TopHat-Fusion, and JAFFA did not detect any fusion candidates with this “smaller data”. SRR1657556 and SRR1657557 represent the “larger data”. All the software packages detected fusion transcripts with this “large data”. Bellerophontes produced more than five thousand fusions with all six runs of this test dataset (Table 4). Of note, there is a small overlap in the fusions detected by various software tools (Supplementary Table S2). This could be due to false discoveries associated with individual software, or the fact that none of the tools are inclusive. Supplementary Table S2 shows the overlap of the fusions between EricScript, FusionCatcher, JAFFA, MapSplice, SOAPfuse, and TopHat-Fusion. These six best tools only have four common fusions among them (Supplementary Table S2).

Previously, we used RT-PCR, and traditional Sanger sequencing to validate 44 fusions from this dataset^10^. Here, we combined all six RNA-Seq analysis results of each fusion detection tool, and compared them to the list of 44 validated fusions. A total of 31, 26, 9, 1, 3, and 5 common fusions were found in the results of Chimerascan, EricScript, FusionHunter, JAFFA, FusionCatcher, and BreakFusion respectively (Supplementary Sheet S2). Other tools did not detect any of the 44 fusions. This observation is consistent with the possibility that none of the tools is inclusive.

**Figure 3:**
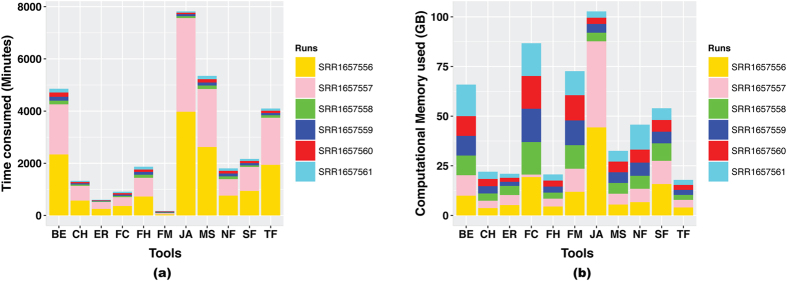
Comparison of computational time and memory used by software packages on the test dataset. (**a**) Times consumed (Minutes) by the software packages to analyse each run of test dataset, (**b**) Computational Memory (GB) used by the software packages to analyse each run of test dataset. **BE**: Bellerophontes, **CH**: Chimerascan, **ER**: EricScript, **NF**: nFuse, **FC**: FusionCatcher, **FH**: FusionHunter, **FM**: FusionMap, **JA**: JAFFA, **MS**: MapSplice, **SF**: SOAPfuse, **TF**: TopHat-Fusion.

In terms of time consumed and memory used, EricScript is again better than other tools (Fig. 3(a,b)). It analyzed SRR1657556, SRR1657557, SRR1657558, SRR1657559, SRR1657560, and SRR165761 data using, 5.253128 GB of RAM for 252 minutes, 5.05028 GB of RAM for 267 minutes, 4.581692 GB for 23 minutes, 16.792396 GB for 49 minutes, 2.02542 GB for 18 minutes, and 2.029776 GB of RAM for 22 minutes respectively (Supplementary Sheet S3). Chimerascan is the second best after EricScript in terms of time consumption and memory utilization (Fig. 3(a,b)). FusionsHunter also showed a promising result with larger data, using 4.520588 GB for 727 minutes and 3.95496 GB for 717 minutes for SRR1657556 and SRR1657557 respectively (Supplementary Sheet S3). When compared to other tools, on the larger data (i.e. SRR1657556 and SRR1657557) JAFFA is the least efficient in terms of time consumption and memory utilization (Fig. 3(a,b)). It required 44.342636 GB for 3,976 minutes, and 43.34852 GB for 3,589 minutes respectively. However, for the smaller data (i.e. SRR1657558, SRR1657559, SRR1657560, and SRR165761) JAFFA is comparable to the other tools, using 4.349008 GB for 72 minutes, 4.421472 GB for 77 minutes, 3.137548 GB for 56 minutes, and 3.15054 GB for 49 minutes respectively (Supplementary Sheet S3). SOAPfuse has a fair balance between time consumption, memory usage, and detected fusions for both large and small data.

## Discussion

The main aim of this study is to assess all of the current fusion detection software packages available to date. We originally planned to evaluate all 20 software tools, and ended up with 12 that were suitable for the study. We analyzed the performances of all of the tools (except for BreakFusion), not only in terms of specificity and sensitivity, but also in terms of computational memory i.e. RAM (Random Access Memory) usage, and time consumed by these tools. In addition to the positive and negative datasets that are publically available, we examined the tools on the mixed and test sets.

The positive dataset contains 57,209 simulated paired-end reads, with 50 true fusion sequences. The fusion reads range from 9 to 8,852. For this small dataset with abundant fusions, JAFFA, EricScript, and MapSplice outperform other tools with a good balance between time consumption, memory usage, and sensitivity.

The negative dataset contains six sets of reads, with varying read length, and quality scores. On this dataset, SOAPfuse and EricScript did not finish the runs for any of the six libraries. Chimeracan only completed the run on Lib_50_R2 data. In terms of the false discovery rate, FusionCatcher was the best, as it did not identify any false fusions. Bellerophontes, Chimerascan, FusionHunter, JAFFA, and TopHat-Fusion detected false fusions only the in case of Lib_50_R2. Lib_50_R2 has a lower quality score when compared to Lib_50_R1^11^, indicating that the quality of short reads plays an important role in chimeric RNA detection. For MapSplice and nFuse, we also noticed a correlation between the quality of short reads and false fusion discovery. There is also a connection between read length and false fusion discovery (Fig. 2). Lib_100 had a higher number of false fusions than Lib_75 for all three tools (MapSplice, nFuse, and FusionMap) (Fig. 2). For the negative dataset, FusionMap used the least amount of time. MapSplice and JAFFA consumed the most time and memory of all of the tools examined (Supplementary Fig. S1).

The mixed dataset mimics a true dataset with some real fusions buried in 75 million reads. Chimerascan and SOAPfuse did not finish the runs for this dataset, due to errors in the intermediate steps. The sensitivity of EricScript (78%) did not differ from its performance in the positive dataset. When comparing their performance on the positive dataset with this mixed dataset, there is an increase in the sensitivity of four tools: Bellerophontes (66% to 68%), FusionMap (56% to 72%), nFuse (30% to 76%), and TopHat-Fusion (54% to 56%). This means that in addition to true fusion reads, these tools also require a certain amount of reads. With this increase in sensitivity, there is also a small increase in the false positive fusion detection rate in the cases of FusionMap (0 to 24), and nFuse (0 to 2). The number of false fusions in the case of Bellerophontes remained the same (i.e. 9). On the other hand, the sensitivity of three tools: FusionCatcher (66% to 62%), JAFFA (88% to 44%), and MapSplice (86% to 84%) dropped. The drastic change in the sensitivity of JAFFA (88% to 44%) is due to complications in the assembly of the negative dataset reads. Misassembles are the leading cause of the poor performance of JAFFA on this mixed dataset. JAFFA also consumed more time and memory on this dataset. EricScript is the best considering that it has the highest PPV, yet the time and memory consumption remained about the same as in the small, positive dataset.

Our test dataset consisted of six real RNA-Seq runs, generated in our previous study^10^. FusionCatcher, FusionMap, JAFFA, and TopHat-Fusion did not produce any fusions in the case of smaller data (i.e. SRR1657558, SRR1657559, SRR1657560, and SRR165761). FusionHunter showed abnormal behavior by predicting a total of 110 and 112 fusions with the larger RNA-Seq runs (i.e. SRR1657556 and SRR1657557), and 236, 238, 230, and 224 fusions in the smaller RNA-Seq runs (Table 4). When compared to the other tools, Bellerophontes predicted the highest number of fusion events in all of the runs of the test dataset (>5000 for all runs). However, since it predicted a total of 15,465 fusions in a negative set (i.e. Lib_50_R2), it is highly likely that a large number of these fusions detected by Bellerophontes are false positives. In contrast, TopHat-Fusion only detected eight and nine fusions in the cases of the larger runs, and did not detect any fusions in the rest of the runs. Even though it had a high PPV, its sensitivity is among the lowest on the mixed dataset. We suspect TopHat-Fusion may miss many true positives. We noticed small overlaps in the fusions detected by various tools. We also compared the detected fusions using each software package with our list of 44 confirmed fusions. A total of 31, 26, 9, 1, 3, and 5 common fusions were found in the results of Chimerascan, EricScript, FusionHunter, JAFFA, FusionCatcher, and BreakFusion respectively (Supplementary Sheet S2). The rest of the tools had no matches using this list of 44 conformed fusions. These results may be partly due to the false discoveries of various tools, but also indicate that none of the fusion detection tools are inclusive. In terms of time and memory used, the performance of EricScript is better than the other tools, consuming less memory and time to analyze the data from all six RNA-Seq runs (Fig. 3). Compared with other tools, JAFFA is the least efficient on larger data. For smaller datasets, JAFFA competes with Chimerascan. However, for smaller data, JAFFA did not detect any fusion candidates (Table 4). Differences in the efficiency of JAFFA with large and small data are clearly seen in the Fig. 3. We used JAFFA in ‘hybrid mode’. This was the combination of its two modes i.e. ‘assembly mode’ and ‘direct mode’. In this mode, it follows four steps; 1) it uses Velvet and Oases to assemble the reads, 2) searches the fusions among the assembled contigs, 3) maps reads to both a known reference transcriptome and the assembled transcriptome, and 4) searches the fusion among the unmapped reads. The complications in the transcriptome assembly in the first step may lead to more memory and time consumption by JAFFA.

**Figure 4:**
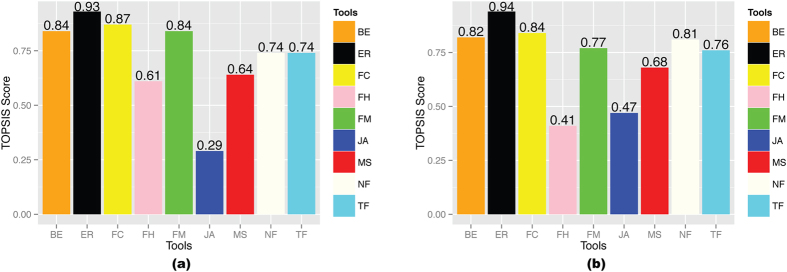
TOPSIS score comparison of the tools. (**a**) TOPSIS scores calculated by giving equal weight to Sensitivity, RAM, Time and PPV, (**b**) TOPSIS scores calculated by giving more weight (i.e. 0.35) to Sensitivity and PPV; and less weight (i.e. 0.15) to RAM and time. **BE**: Bellerophontes, **ER**: EricScript, **FC**: FusionCatcher, **FH**: FusionHunter, **FM**: FusionMap, **JA**: JAFFA, **MS**: MapSplice, **NF**: nFuse, **TF**: TopHat-Fusion.

TOPSIS analysis was performed for the final ranking of the fusion detection tools. This analysis was performed on the mixed dataset. We ranked the tools on the basis of TOPSIS score, which was calculated in two scenarios. In the first scenario, we have equal weights for sensitivity, time, RAM and PPV (i.e. 0.25 each) (Supplementary Sheet S4). In the second scenario, we put more weight on the sensitivity and PPV (0.35 each), and less weight on time and RAM (0.15 each) (Supplementary Sheet S4). In the first situation, the ranking of the tools is EricScript > FusionCatcher > FusionMap > Bellerophontes > TopHat-Fusion > nFuse >MapSplice > FusionHunter > JAFFA (Fig. 4(a)). In the second situation, they are ranked as EricScript > FusionCatcher > Bellerophontes > nFuse > FusionMap > TopHat-Fusion > MapSplice > JAFFA > FusionHunter (Fig. 4(b)). In both cases, the TOPSIS score of EricScript is the highest. SOAPfuse and Chimerascan were not able to finish the run on this mixed dataset, so are not included in this analysis. However, based on their performances in other datasets, they are not superior to EricScript.

In conclusion, we have evaluated the performance of all of the tools that are currently available, and suitable for this type of analysis. Among them, we found that EricScript had 100% PPV on the mixed dataset. This software detected a reasonable number of fusions, with a sensitivity of 78%. EricScript was also shown to require the least amount of time and memory utilization. We also found that although some of the most recent tools, such as JAFFA and SOAPfuse have features that appear to give them the advantage over the older tools, they require more time consumption and computational memory usage. In addition, the performances of 12 tools on sensitivity, specificity, and efficiency (time and computational memory usage) differ among different datasets. The performances of some tools also changed depending on the RNA-Seq read length, read number, and the quality of the reads. Users should choose the best tool fitting their needs, based on the properties of their RNA-Seq datasets.

## Additional Information

**How to cite this article**: Kumar, S. *et al.* Comparative assessment of methods for the fusion transcripts detection from RNA-Seq data. *Sci. Rep.* **6**, 21597; doi: 10.1038/srep21597 (2016).

## Supplementary Material

Supplementary Information

Supplementary Sheet S1

Supplementary Sheet S2

Supplementary Sheet S3

Supplementary Sheet S4

## Figures and Tables

**Table 1:** A complete summary of 12 fusion-detection tools.

Tool Name	Group	Reference	URL	Year
Bellerophontes	Paired-end + Fragmentation	Bioinformatics. (Abate *et al.*^25^)	http://eda.polito.it/bellerophontes/	2012
BreakFusion	Whole paired-end	Bioinformatics. (Chen *et al.*^18^)	http://bioinformatics.mdanderson.org/main/BreakFusion	2012
ChimeraScan	Paired-end + Fragmentation	Bioinformatics. (Iyer *et al.*^29^)	http://code.google.com/p/chimerascan/	2011
EricScript	Whole paired-end	Bioinformatics. (Benelli *et al.*^22^)	http://sourceforge.net/projects/ericscript/	2012
FusionCatcher	Paired-end + Fragmentation	bioRxiv. (Nicorici *et al.*^23^)	http://code.google.com/p/fusioncatcher/	2012
FusionHunter	Whole paired-end	Bioinformatics. (Li *et al.*^12^)	http://bioen-compbio.bioen.illinois.edu/FusionHunter/	2011
FusionMap	Direct Fragmentation	Bioinformatics. (Ge *et al.*^13^)	http://www.arrayserver.com/wiki/index.php?title=FusionMap	2011
JAFFA	Paired-end + single-end	Genome Medicine. (Davidson *et al.*^20^)	https://github.com/Oshlack/JAFFA/wiki	2015
MapSplice	Direct Fragmentation	Nucleic Acids. Research (Wang *et al.*^15^)	http://www.netlab.uky.edu/p/bioinfo/MapSplice	2010
nFuse	Whole paired-end	Genome research. (McPherson *et al.*^21^)	https://code.google.com/p/nfuse/	2012
SOAPFuse	Whole paired-end	Genome biology. (Jia *et al.*^19^)	http://soap.genomics.org.cn/soapfuse.html	2013
TopHat-Fusion	Paired-end + Fragmentation	Genome biology. (Kim and Salzberg, 2011)	http://tophat.cbcb.umd.edu/fusion_index.html	2011

**Table 2:** Performances of all 12 tools on the positive dataset.

Tools	True Fusions detected	Sensitivity (%)	Time used (Minutes)	Memory (GB)
Bellerophontes	33(42)	66	6	9.25
BreakFusion	2	4	–	–
Chimerascan	4	8	3	2.75
EricScript	39	78	3	0.23
FusionCatcher	33	66	11	11.67
FusionHunter	18	36	9	2.31
FusionMap	28	56	19	11.84
JAFFA	44	88	7	4.30
MapSplice	43	86	3	5.35
nFuse	15	30	46	12.59
SOAPfuse	41	82	52	7.61
TopHat-Fusion	26	54	15	2.32

This dataset contained a total of 50 fusions. Sensitivity is the percentage of true fusions detected out of 50. Out of 42 fusions produced by Bellerophontes, 33 are the true fusions. For other tools, all of the fusions produced were true fusions.

**Table 3:** Performance of fusion-detection tools on the mixed dataset.

Tools	Total Fusions detected	True fusions detected	False fusions detected	Sensitivity (%)	Positive predictive value (%)	Time used (Minutes)	Memory (GB)
Bellerophontes	43	34	9	68	79	1012	10.38
BreakFusion	*	*	*	*	*	*	*
Chimerascan	*	*	*	*	*	*	*
EricScript	39	39	0	78	100	677	4.67
FusionCatcher	31	31	0	62	100	932	1.76
FusionHunter	0	0	0	–	–	1202	5.86
FusionMap	60	36	24	72	60	120	12.50
JAFFA	23	22	1	44	95.6	3845	89.4
MapSplice	77	42	35	84	54	3825	5.48
nFuse	40	38	2	76	95	2306	12.57
SOAPfuse	*	*	*	*	*	*	*
TopHat-Fusion	28	28	0	56	100	2443	2.55

^*^Indicates the software errors occurred in the handling of intermediate files. No final result was produced.

**Table 4:** Number of fusions detected in the test dataset.

Tools	SRR1657556	SRR1657557	SRR1657558	SRR1657559	SRR1657560	SRR1657561
Bellerophontes	5119 (5112)	5034 (5029)	5695 (5694)	5284 (5281)	5580 (5579)	5241 (5239)
BreakFusion	926 (861)	992 (924)	407 (400)	420 (416)	380 (373)	428 (427)
Chimerascan	292 (275)	293 (286)	23 (21)	38 (37)	31 (31)	27 (25)
EricScript	259 (259)	324 (324)	7 (7)	7 (7)	4 (4)	10 (10)
FusionCatcher	9 (7)	20 (18)	0	0	0	0
FusionHunter	110 (84)	112 (90)	236 (145)	238 (143)	230 (144)	224 (130)
FusionMap	11	15	0	0	0	0
JAFFA	252 (250)	252 (251)	0	0	0	0
MapSplice	145	108	13	11	15	7
nFuse	92 (88)	99 (97)	2 (2)	6	5	3
SOAPfuse	56 (53)	55 (51)	3 (2)	4 (3)	5 (4)	7 (6)
TopHat-Fusion	8	9 (8)	0	0	0	0

The numbers in the brackets indicate the unique gene pairs.
