# Supplementary material for: Comparative assessment of methods for the fusion transcripts detection from RNA-Seq data
Source: Sci Rep. 2016 Feb 10;6:21597. doi: 10.1038/srep21597 (PMC4748267; doi:10.1038/srep21597)
Supplement: Supplementary Information [file srep21597-s1.pdf]

Comparative assessment of methods for the fusion transcripts detection from RNA-Seq data

Shailesh Kumar[1], Angie Duy Vo[1], Fujun Qin[1], Hui Li[1,2,*]

[1]Department of Pathology, School of Medicine, University of Virginia, Charlottesville, VA 22908

[2]Department of Biochemistry and Molecular Genetics, School of Medicine, University of Virginia, Charlottesville, VA 22908

[*]Corresponding author.

E-mail: hl9r@virginia.edu

Web: http://lilab.medicine.virginia.edu

Phone: +1- 434-9826680

Fax: +1-434-2437244

Running title: Benchmarking software tools for fusion transcript detection

Keywords: fusion transcript, chimeric RNA, RNA-Seq,

Supplementary Information

List of Tables

Supplementary Table S1: False discoveries for the 12 tools. The total numbers of fusions detected on the negative dataset are listed. Numbers in the brackets indicate the unique gene pairs. * Indicates that the software errors occurred in the handling of intermediate files, no final result was produced.

Supplementary Table S2: Number of overlapping fusions between all the 12 tools on the test dataset. **BE**: Bellerophontes, **BF:** BreakFusion, **CH**: Chimerascan, **ER**: EricScript, **NF**: nFuse, **FC**: FusionCatcher, **FH**: FusionHunter, **FM**: FusionMap, **JA**: JAFFA, **MS**: MapSplice, **SF**: SOAPfuse, **TF**: TopHat-Fusion.

List of Figures

Supplementary Figure S1: Comparison of time and computational memory used by software packages on the negative dataset. (a) Lib_50_R1, (b) Lib_50_R2, (c) Lib_75_R1, (d) Lib_75_R2, (e) Lib_100_R1, (f) Lib_100_R2. **BE**: Bellerophontes, **CH**: Chimerascan, **ER**: EricScript, **NF**: nFuse, **FC**: FusionCatcher, **FH**: FusionHunter, **FM**: FusionMap, **JA**: JAFFA, **MS**: MapSplice, **SF**: SOAPfuse, **TF**: TopHat-Fusion.

Supplementary Figure S2: Comparison of time and computational memory, used by software packages on the mixed dataset. **BE**: Bellerophontes, **ER**: EricScript, **NF**: nFuse, **FC**: FusionCatcher, **FH**: FusionHunter, **FM**: FusionMap, **JA**: JAFFA, **MS**: MapSplice, **TF**: TopHat-Fusion.

Supplementary Figure S3: Overlapping and unique fusions among the six best tools on the test dataset. **ER**: EricScript, **FC**: FusionCatcher, **JA**: JAFFA, **MS**: MapSplice, **SF**: SOAPfuse, **TF**: TopHat-Fusion.

List of Sheets

Supplementary Sheet S1: Number of detected fusions, time and memory used by different tools on negative dataset.

Supplementary Sheet S2: A list of conformed fusions and common fusions detected by other tools on the test dataset.

Supplementary Sheet S3: Number of detected fusions, time and memory used by different tools on the test dataset.

Supplementary Sheet S4: TOPSIS analysis and calculation of final scores for the fusion detection tools on the mixed dataset.

Supplementary Table S1: False discoveries for the 12 tools. The total numbers of fusions detected on the negative dataset are listed. Numbers in the brackets indicate the unique gene pairs. * Indicates that the software errors occurred in the handling of intermediate files, no final result was produced.

Tools	Lib_50_R1	Lib_50_R2	Lib_75_R1	Lib_75_R2	Lib_100_R1	Lib_100_R2
**Bellerophontes**	0	15465 (15430)	0	0	0	0
**BreakFusion**	*	*	*	*	*	*
**Chimerascan**	*	1600 (1540)	*	*	*	*
**EricScript**	*	*	*	*	*	*
**FusionCatcher**	0	0	0	0	0	0
**FusionHunter**	0	842 (767)	0	0	0	0
**FusionMap**	93	27	24	27	81	83
**JAFFA**	0	51(48)	0	0	0	0
**MapSplice**	0	152	33	30	45	42
**nFuse**	5	70(67)	39 (35)	31 (29)	54 (53)	78 (70)
**SOAPfuse**	*	*	*	*	*	*
**TopHat-Fusion**	0	29 (25)	0	0	0	0

Supplementary Table S2: Number of overlapping fusions between the 12 tools on the test dataset. **BE**: Bellerophontes, **BF:** BreakFusion, **CH**: Chimerascan, **ER**: EricScript, **NF**: nFuse, **FC**: FusionCatcher, **FH**: FusionHunter, **FM**: FusionMap, **JA**: JAFFA, **MS**: MapSplice, **SF**: SOAPfuse, **TF**: TopHat-Fusion.

**Tools**	**BE**	**BF**	**CH**	**ER**	**FC**	**FH**	**FM**	**JA**	**MS**	**NF**	**SF**	**TF**
**BE**	1820	0	0	2	0	0	0	0	0	2	1	0
**BF**	0	1401	5	4	0	173	1	2	2	1	3	0
**CH**	0	5	463	43	9	5	7	14	7	1	42	5
**ER**	2	4	43	474	14	1	9	13	8	1	38	6
**FC**	0	0	13	14	19	0	11	14	11	1	19	9
**FH**	0	173	5	1	0	287	0	0	0	1	2	0
**FM**	0	1	7	9	7	0	16	10	9	1	8	5
**JA**	0	2	14	13	9	0	10	485	10	1	12	6
**MS**	0	2	7	8	7	0	9	10	25	2	8	4
**NF**	2	1	1	1	1	1	1	1	2	150	1	2
**SF**	1	3	42	38	14	2	8	12	8	1	85	6
**TF**	0	0	5	6	5	0	5	6	4	2	6	11

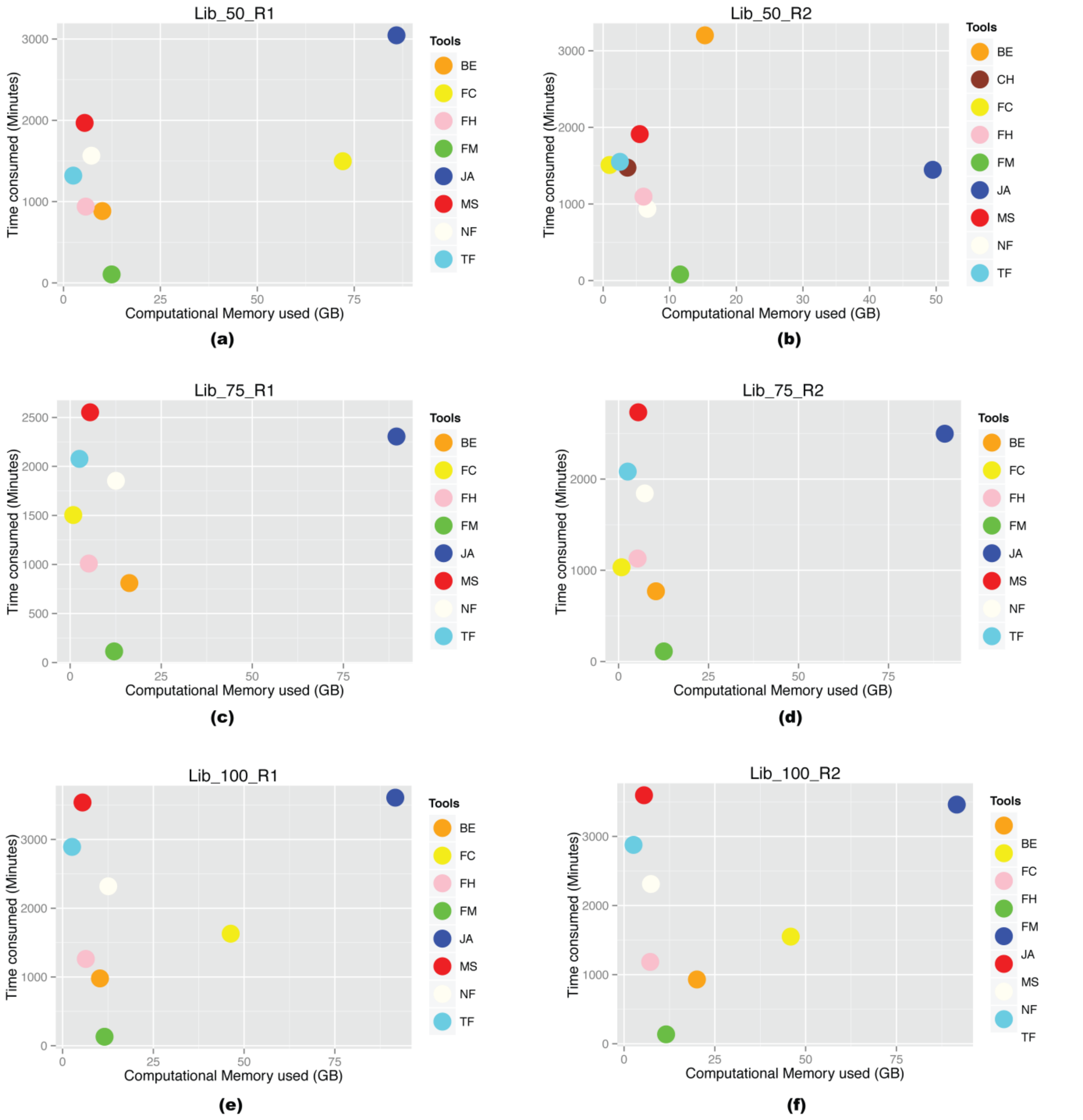

Supplementary Figure S1: Comparison of time and computational memory used by software packages on the negative dataset. (a) Lib_50_R1, (b) Lib_50_R2, (c) Lib_75_R1, (d) Lib_75_R2, (e) Lib_100_R1, (f) Lib_100_R2. **BE**: Bellerophontes, **CH**: Chimerascan, **ER**: EricScript, **NF**: nFuse, **FC**: FusionCatcher, **FH**: FusionHunter, **FM**: FusionMap, **JA**: JAFFA, **MS**: MapSplice, **SF**: SOAPfuse, **TF**: TopHat-Fusion.

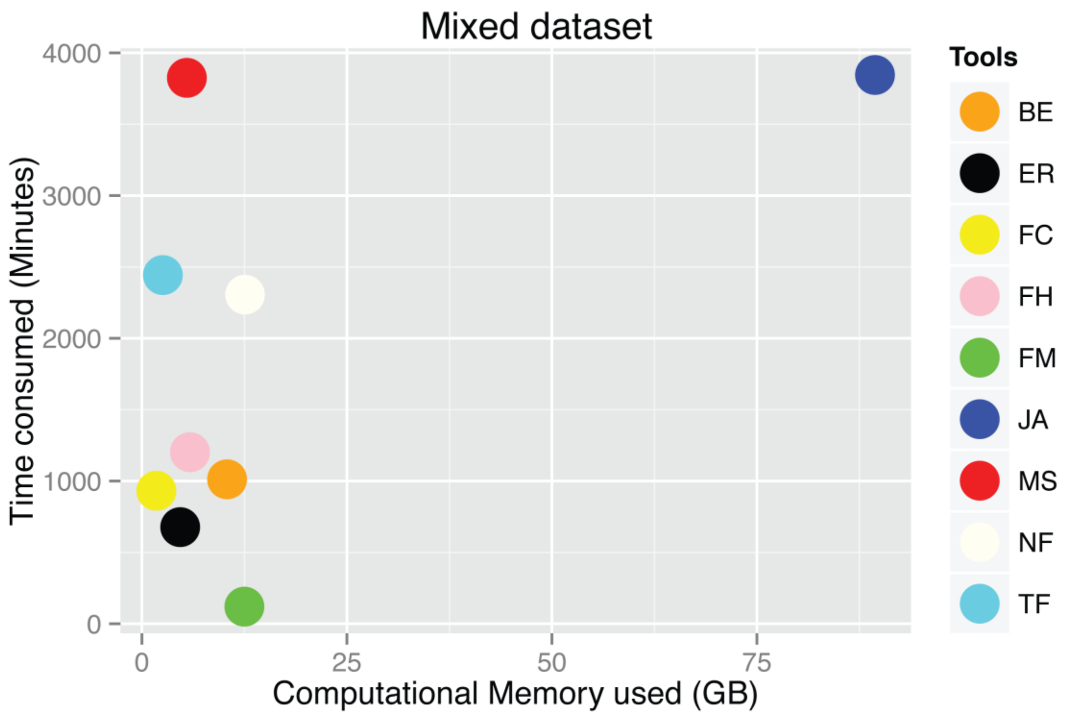

Supplementary Figure S2: Comparison of time and computational memory, used by software packages on the mixed dataset. BE: Bellerophontes, **ER**: EricScript, **NF**: nFuse, **FC**: FusionCatcher, **FH**: FusionHunter, **FM**: FusionMap, **JA**: JAFFA, **MS**: MapSplice, **TF**: TopHat-Fusion.

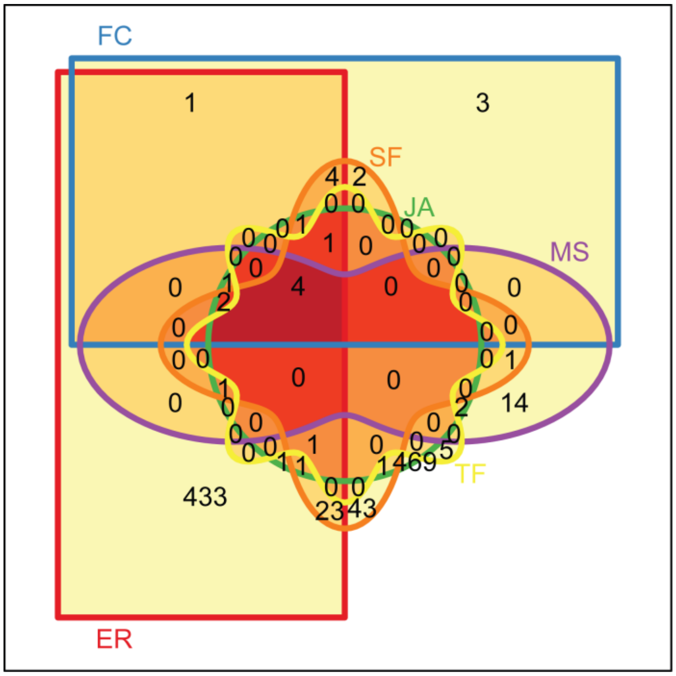

Supplementary Figure S3: Overlapping and unique fusions among the six best tools on the test

dataset. **ER**: EricScript, **FC**: FusionCatcher, **JA**: JAFFA, **MS**: MapSplice, **SF**: SOAPfuse, **TF**: TopHat-Fusion.
